# Plasma soluble TIM-3 is increased in normoglycemic South Asian women compared to Nordic women after gestational diabetes mellitus and associated with markers of metaflammation

**DOI:** 10.1016/j.heliyon.2024.e40339

**Published:** 2024-11-16

**Authors:** Helene Grannes, Archana Sharma, Anita Suntharalingam, Annika E. Michelsen, Pål Aukrust, Thor Ueland, Kåre I. Birkeland, Ida Gregersen, Sindre Lee-Ødegård, Bente Halvorsen

**Affiliations:** a Research Institute for Internal Medicine, Oslo University Hospital, Oslo, Norway; b Institute of Clinical Medicine, University of Oslo, Oslo, Norway; c Department of Endocrinology, Akershus University Hospital, University of Oslo, 1478, Lørenskog, Norway; d Dep. of Endocrinology, Morbid Obesity and Preventive Medicine, Oslo University Hospital, Oslo, Norway; e Section of Clinical Immunology and Infectious Diseases, Oslo University Hospital, Oslo, Norway; f Thrombosis Research and Expertise Centre, University of Tromsø, Tromsø, Norway; g Division of Cardiovascular Medicine, Department of Medicine, Brigham and Women's Hospital, Harvard Medical School, Boston, MA, USA

**Keywords:** T -cell, sTIM-3, Immune cells, Ethnicity, Obesity, Type 2 diabetes

## Abstract

**Background:**

Women with South Asian ethnicity have a higher risk of developing type 2 diabetes mellitus (T2DM) compared with white women of European descent, especially after gestational diabetes mellitus (GDM). Central obesity and adipose tissue dysfunction have been linked to their higher risk of T2DM, but the mechanisms are not known. We hypothesize that low-grade, persistent immune cell activation is involved in metabolic disturbances following GDM with different influence according to ethnicity.

**Methods:**

We measured plasma levels of T cell exhaustion *marker soluble T cell immunoglobin mucin domain 3* (sTIM-3), sCD25, sCD27 and soluble lymphocyte activation gene (sLAG)-3 in 266 women of South Asian (n = 160) and white Nordic (n = 106) ethnic background with a history of GDM.

**Results:**

Baseline plasma concentration of sTIM-3 was higher in South Asian women compared to Nordic women (p < 0.001). This difference was driven by higher sTIM-3 in South Asian women with NGT, compared to their Nordic counterparts (p = 0.005) but there were no significant differences comparing Nordic and South Asian women with altered glucose tolerance (AGT). Soluble TIM-3 correlated positively with waist-height ratio (WHtR) and body mass index across all groups, but whereas sTIM-3 correlated moderately and consistently with markers of metaflammation in South Asians, this pattern was not found in Nordic women. Mediation analysis indicated that 15 % of the difference found in adipose insulin resistance between ethnicities could be mediated by sTIM-3, and that 33 % of the difference in sTIM-3 concentrations could be mediated by WHtR. Moreover, T cell markers sCD27 and sLAG3 were also increased in South Asian women compared with Nordic women, further supporting involvement of T cell activation in these women.

**Conclusion:**

We found increased levels of sTIM-3, as well as additional markers of T cell activation/exhaustion, in a population of normoglycemic South Asian women with previous gestational diabetes as compared to women of Nordic descent. The possible causal relationship between T cell activation and metabolic dysfunction in high-risk South Asian women is however still elusive and merits further investigation.

## Highlights

• Normoglycemic South Asian women had increased plasma levels of sTIM-3 as compared to Nordic women after gestational diabetes.
• In South Asian women, sTIM-3 correlated with markers of metaflammation, a pattern that was not found in Nordic women.
• South Asian women had increased calculated adipose insulin resistance compared to Nordic women.
• Mediation analysis indicated that 16 % of the difference observed in adipose tissue resitance could be mediated by sTIM-3.

## 1 Introduction

Women of South Asian ethnicity have a substantially higher risk of developing type 2 diabetes (T2DM) than white European women, especially after gestational diabetes mellitus (GDM) [1,2]. Therefore, studying a cohort of women diagnosed with GDM during a previous, recent pregnancy, it is likely that a proportion of these women are, by the time of the study, in the process of developing T2DM, allowing research into the developing phase and the changes occurring before the debut of T2DM. We have previously shown that South Asian women exhibit early signs of metabolic dysfunction, even when classified as normoglycemic, suggesting a faster development of insulin resistance as compared to Nordic women [3]. The mechanisms behind these differences in relation to ethnicity, are however, not completely understood.

Increased central obesity in South Asian individuals has been linked to their higher risk of T2DM [4], and adipose tissue dysfunction is thought to be an important driver of insulin resistance, especially in South Asian women [5], and central obesity is associated with adiposopathy or “sick fat” [6]. Metabolic disturbances and immune activation is tightly linked, and T2DM is characterized by chronic low-grade inflammation triggered by metabolic dysfunction, termed metaflammation [7]. The chain of events leading from increase in adipose tissue to insulin resistance is, however, not yet fully elucidated, but T cells seem to play a pathogenic role [8]. It has been observed that mice with reduced numbers of pro-inflammatory Th1 cells have reversed obesity-associated insulin resistance, suggesting a causal relationship between T cell-driven immune activation and disturbed insulin sensitivity [9], but the mechanisms in humans are less clear. To further illustrate the complexity of these interactions, research has shown that the insulin receptor plays a previously underappreciated role as a T cell activator [10]. Thus, increases in circulating insulin as well as inflammatory cytokines related to altered insulin sensitivity could provide constant stimuli to T cells, even in early stages of T2DM. Therefore, insulin resistance, followed by increase in insulin secretion may in turn affect T cell activity, contributing to a vicious state of persistent T cell activation and potentially T cell exhaustion. T *cell immunoglobulin and mucin domain 3* (TIM-3) is considered an exhaustion marker for T cells [11,12], although it is also expressed on other cells such as NK cells, macrophages, and dendritic cells. TIM-3 can be cleaved from the cell surface by certain matrix metalloproteinases to a soluble form [13], and although the function(s) of soluble TIM-3 (sTIM-3) is not clarified, it has been reported to correlate to the degree of membrane expression of TIM-3 and has been suggested as an attractive soluble marker of persistent T cell activation and exhaustion in various disorders [14]. Increased expression of TIM-3 is observed in T cells in obesity, and in T cells and NK cells in T2DM [[15], [16], [17], [18]]. The secretion of sTIM-3 during altered glucose tolerance (AGT) is, however, largely unknown. We hypothesize that low-grade, persistent T cell activation is involved in the development of metabolic disturbances, with differences according to ethnicity. Herein, we measured plasma levels of sTIM-3, as well as sLAG-3, sCD25 and sCD27, as a proxy of persistent T cell activation/exhaustion in a population of South Asian and Nordic women diagnosed with GDM in their last pregnancy and thus at high risk of developing T2DM.

## 2 Materials and methods

### 2.1 Study population

The design, recruitment, and data collection are detailed elsewhere [3]. In brief, women with South Asian and Nordic ethnicity with a history of GDM during a pregnancy 12–36 months prior to the study, as identified from their medical records at three major hospitals in the Oslo region, were invited to participate. In brief, GDM diagnosis was set according to the WHO 1999 or modified International Association of Diabetes and Pregnancy Study Group (IADPSG) criteria, as previously described [3,19]. Demographic characteristics are shown in Table 1. The inclusion period lasted from September 2018 to December 2021. The inclusion criteria were age ≥18 years and ethnic origin from (both parents born in) a South Asian (Pakistan, India, Sri Lanka and Bangladesh) or a Nordic country (Norway, Sweden, Denmark, Finland and Iceland). Exclusion criteria were new pregnancies after the index pregnancy, exclusive breastfeeding at the time of examination, known diabetes, ongoing inflammatory disease or serious co-morbidity, or a history of major surgical procedure <3 months prior to inclusion. At the study visit, height and weight were measured, as well as waist and hip circumferences. All women did an oral glucose tolerance test (OGTT) after at least 8 h fasting. Glycaemic status was defined as normal glucose tolerance (NGT) if the fasting plasma glucose (FPG) was <6.1 mmol/L, and HbA1c < 42 mmol/mol, and 2 h plasma glucose <7.8 mmol/L [20]. All other combinations of results were defined as hyperglycaemic and grouped as prediabetes and diabetes, hereafter termed “altered glucose tolerance” (AGT) [20]. The study protocols were approved by the Regional Committees for Medical and Health Research Ethics, reference number: 2018/689. The study confirms the principles outlined in the Declaration of Helsinki for use of human samples or subjects. Signed informed consent was obtained from all participants.

**Table 1:** Participant characteristics from the DIASA1 study.

	All			Normal Glucose Tolerance			Altered Glucose Tolerance		
	Nordic	South Asian	p	Nordic	South Asian	p	Nordic	South Asian	p
**Age (years)**	36.6 (4.9)	34.5 (4.1)	<0.001	36.8 (4.9)	34.1 (3.9)	0.002	36.3 (4.9)	34.6 (4.3)	0.032
**BMI (kg/m**^**2**^**)**	29.3 (6.8)	29.1 (5.3)	0.758	26.9 (6.9)	28.4 (6.1)	0.263	32.3 (5.7)	29.4 (5.0)	0.004
**WHtR**	0.58 (0.1)	0.61 (0.1)	0.002	0.6 (0.1)	0.7 (0.2)	0.004	0.6 (0.1)	0.6 (0.16)	0.649
**Years since index preg.**	1.7 (0.6)	1.6 (0.7)	0.289	1.7 (0.6)	1.7 (0.7)	0.980	1.8 (0.7)	1.6 (0.6)	0.154
**HbA1c preg. (mmol/mol)**	37.5 (4.3)	40.1 (4.5)	<0.001	36.3 (4.9)	38.4 (3.6)	0.025	38.5 (3.6)	40.8 (4.6)	0.003
**Family DM, yes %**^a^	21 (24)	112 (75)	<0.001	10 (45)	35(80)	<0.001	11 (25)	77 (73)	<0.001
**HbA1c visit (mmol/mol)**	36.5 (4.0)	39.2 (4.6)	<0.001	35.0 (2.5)	36.8 (3.3)	0.003	38.1 (4.6)	40.3 (4.7)	0.006
**Insulin (pmol/L)**	76.4 (47.0)	120.3 (65.4)	<0.001	60.8 (35.6)	98.1 (48.4)	<0.001	94.6 (52.2)	129.5 (69.3)	0.002
**HOMA2-S**	91.5 (50.2)	57.4 (32.9)	<0.001	111.4 (54.1)	68.5 (38.8)	<0.001	67.7 (31.9)	52.8 (29.1)	0.005
**HOMA2β**	93.5 (39.4)	119.4 (43.5)	0.001	87.6 (33.2)	116.6 (41.9)	0.001	100.0 (44.7)	120.6 (44.3)	0.008
**IGI**^b^	0.9 (0.5–1.5)	1.0 (0.7–1.7)	0.066	1.0(0.7–1.5)	1.4 (1.0–2.6)	0.004	0.8 (0.4–1.4)	0.9 (0.6–1.4)	0.193
**DI**	4.4 (3.3)	3.3 (2.5)	0.001	6.1 (3.6)	5.3 (2.9)	0.177	2.5 (1.1)	2.5 (1.7)	0.982
**Matsuda index**	4.7 (2.6)	2.8 (1.8)	<0.001	5.9 (2.7)	3.5 (2.4)	<0.001	3.4 (1.7)	2.5 (1.5)	0.001
**NEFA (mq/L)**	0.5 (0.2)	0.5 (0.2)	0.488	0.5 (0.2)	0.5 (0.2)	0.138	0.6 (0.2)	0.6 (0.2)	0.281
**TG (mmol/L)**	1.0 (0.7)	1.3 (0.7)	0.002	0.8 (0.3)	1.1 (0.6)	<0.001	1.3 (0.8)	1.4 (0.7)	0.596
**CRP**^b^**(mg/L)**	1.2 (0.5–4.3)	2.1 (1.1–4.3)	0.005	0.8 (0.3–2.2)	1.6 (0.8–3.2)	0.007	2.2 (0.9–5.8)	2.5 (1.3–4.5)	0.901
**IL-6**^b^**(ng/L)**	1.5 (1.5–2.4)	2.3 (1.5–3.2)	<0.001	1.5 (1.5–1.9)	2.3 (1.6–3.2)	<0.001	1.9 (1.5–3.1)	2.3 (1.5–3.3)	0.147
**Leptin (pmol/L)**	1618.6 (1123.4)	1889.1 (980.0)	0.048	1247.64 (977.1)	1718.1 (765.2)	0.012	2043.8 (1138.5)	1962.2(1053.7)	0.67
**Adiponectin (mg(ml)**	10.5 (5.4)	6.7 (3.9)	<0.001	11.5 (5.9)	6.4 (3.5)	<0.001	9.3 (4.7)	6.8 (4.1)	<0.001
**AT-IR**	40.6 (31.0)	65.3 (40.7)	<0.001	29.8 (24.5)	53.3 (36.8)	<0.001	53.2 (33.2)	70.2 (41.3)	0.014

Data are mean and (standard deviation).

a Numbers (percent, %), or.

b Median and [q25-q75] or as indicated. BMI = body mass index, WHtR = waist-height ratio, DM = diabetes mellitus, HOMA = The Homeostasis Model Assessment of beta cell function, IGI = insulinogenic index, DI = disposition index, NEFA = non-esterified fatty acids, TG = triglycerides, CRP = C reactive protein, IL-6 = interleukin 6, AT-IR = Adipose insulin resistance index. P = P-value from Wilcoxon's rank test, or Student *t-*test, as appropriate.

### 2.2 Blood sampling and plasma analysis

Blood was collected in 4 mL EDTA tubes, centrifuged at 3040 g for 10 min at 4 °C, and plasma and serum were stored at −80 °C until analysis. Plasma levels of sTIM-3, sCD25, sCD27 and soluble slymphocyte activation gene (LAG)-3 were measured by enzyme immunoassay obtained from R&D Systems (Minneapolis, Minnesota, USA). Serum or plasma levels of glucose, insulin, triglycerides (TG), non-esterified fatty acids (NEFA) were analyzed at Division of Laboratory Medicine, Oslo University Hospital, Oslo, Norway. Serum leptin levels were measured using an enzyme linked immunosorbent assay (ELISA) kit (Mediagnost). Serum adiponectin levels were measured using a radioimmunoassay kit (Merck Millipore). Plasma high-sensitive C-reactive protein (hsCRP) levels were measured with an Elecsys assay (Roche Diagnostics). Plasma interleukin-6 (IL-6) levels were measured using the Elecsys IL-6 immunoassay kit (Roche Diagnostics).

### 2.3 Calculation of indexes

The adipose tissue insulin resistance index (AT-IR) was calculated as: fasting insulin (pmol/L) x fasting NEFA concentrations (mmol/L) [5,21]. The Homeostasis Model Assessment (HOMA) of insulin sensitivity (HOMA2-S) was calculated from fasting insulin (pmol/L) together with FPG (mmol/L) and HOMA of beta cell function (HOMA2-β) was calculated from fasting serum C-peptide (pmol/L) together with FPG (mmol/L) using the HOMA calculator [22].

Insulinogenic index (IGI) was estimated with the formula: Δinsulin_0–30 min_ (μIU/mL)/Δglucose_0–30 min_ (mg/dL) [23]. The whole-body insulin sensitivity was estimated by the Matsuda index of insulin secretion (ISI) as 10,000/√(fasting serum insulin [μIU/mL] × FPG [mg/dL]) × (mean OGTT insulin [μIU/mL]) × (mean OGTT glucose [mg/dL]) [24]. Finally, Disposition index (DI) was calculated as the product of IGI x Matsuda ISI [25]. Full explanation of these indexes has been previously described [3].

### 2.4 Statistics

For missing values in the data set we omitted the participant in question on an analysis-to-analysis basis. Of all measurements done of the markers, only one value was found so extreme as to be excluded, and this value was set as missing. For testing variables with normal or approximate normal distribution (skewedness <2.0), an independent *t*-test was used. For non-normally distributed data, we used U Mann Whitney. We used Chi-test for comparison of categorical variables. For the correlation analysis, we used the Spearman Rho coefficient with two tailed significances. Variables included in the correlation analysis were selected based on biological relevance, and the main criteria was a direct effect on T cell activity and/or significant difference between the two ethnicities as well as relevance to our hypothesis. We performed mediation analysis with the *regmedint* package, assuming there was no exposure-mediator interaction and that the mediators have preceded the outcome, even though mediator and outcome was measured at the same time [26,27]. P < 0.05 was regarded as statistically significant. For statistical analyses and figures we used IBM SPSS Statistics v. 29.0.0.0 (241), RStudio v.1.4.17171 with R v.4.1.3 and GraphPad Prism 9.

## 3 Results

### 3.1 Study population

The cohort in our study, consisted of 266 individuals, 106 Nordic and 160 South Asian women with previously diagnosed GDM. At baseline the groups had similar BMI and fasting glucose levels, but South Asian women had higher waist-height ratio (WHtR), HbA1c, insulin, IL-6, CRP and a higher calculated AT-IR and HOMA-2 β, and lower adiponectin levels, HOMA2-S, disposition index (DI) and Matsuda index (Table 1, some variables previously described in Refs. [3,28]). Based on the results from OGTT and HbA1c (see Methods for definition), 30 % of the South Asian (n = 48) and 53 % of the Nordic women (n = 56) were classified as having a normal glucose tolerance (NGT) (p < 0.001).

### 3.2 Increased plasma concentrations of T cell marker sTIM-3 in South Asian women after gestational diabetes

**Fig. 1:**
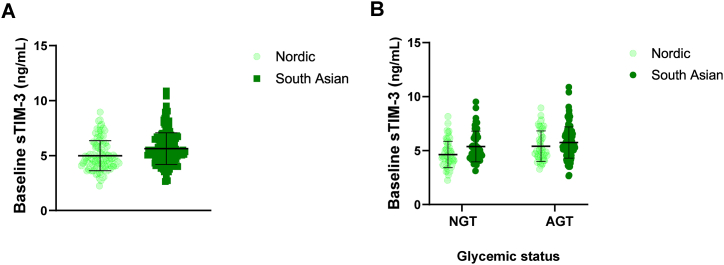
**A)** Plasma concentrations of sTIM3 at baseline for Nordic (light green, n = 106) and South Asian (dark green, n = 160) women with previous gestational diabetes. **B)** Plasma concentrations of sTIM3 at baseline for Nordic (light green) and South Asian (dark green) women with previous gestational diabetes separatet by glycemic status; normoglycemic women (NGT, left, n = 56 Nordic and n = 48 South Asian women) and women with AGT (AGT, right, n = 50 Nordic and n = 112 South Asian women). Presented as Violin plots with mean/SD as boxplots. ∗∗p < 0.01, ∗∗∗p < 0.001. (For interpretation of the references to colour in this figure legend, the reader is referred to the Web version of this article.)

Baseline plasma levels of sTIM-3 were higher in South Asian compared to Nordic women (p < 0.001) (Fig. 1A). When dividing the women according to glycemic status, baseline sTIM-3 was higher in South Asian than in Nordic women with NGT (p = 0.005), while there were no differences when comparing Nordic and South Asian women with AGT (p = 0.169) (Fig. 1B). After adjusting for WHtR in the full cohort, the difference in sTIM3 between ethnicities remained statistically significant (p = 0.013), while when adjusting for insulin this difference was non-significant (p = 0.073). In the NGT group, the difference was lost after adjusting for WHtR (p = 0.092), however not when adjusting for insulin (p = 0.039). Importantly, differences in sTIM3 between ethnicities remained statistically significant after adjusting for AT-IR, both in the population as a whole (p = 0.031) and in the NGT group (p = 0.025), as well as with adjustment for DI, however only in the group as a whole (p = 0.004 and p = 0.068, respectively).

### 3.3 Soluble TIM-3 in relation to waist-height ratio, BMI and adipose tissue insulin resistance

Soluble TIM-3 correlated positively with WHtR in the full cohort (r_ALL_ = 0.35, p < 0.001), in the two ethnic groups evaluated separately (r_N_ = 0.34, p < 0.001; r_SA_ = 0.30, p < 0.001) and across glycemic status (r_NGT_ = 0.39, p < 0.001; r_AGT_ = 0.22, p = 0.006) (Table 2). When dividing the cohort in groups based on ethnicity *and* glycemic status, the positive correlation remained significant in the South Asian women (r_SA-NGT_ = 0.43, p = 0.002; r_SA-AGT_ = 0.20, p = 0.039), but not for the Nordic women (r_N-NGT_ = 0.24, p = 0.08; r_N-AGT_ = 0.20, p = 0.17). The correlation pattern between sTIM-3 and BMI mirrored that of sTIM-3 and WHtR (Table 2). Further, AT-IR correlated positively with sTIM-3 in all Nordic groups (r_N_ = 0.36, p=<0.001; r_N-NGT_ = 0.25, p = 0.065; r_N-AGT_ = 0.26, p = 0.078) whereas South Asian women presented correlations in the full group (r_SA_ = 0.20, p = 0.012) and in the group with AGT (r_SA-AGT_ = 0.28, p = 0.004), but not in the group with NGT (r_SA-NGT_ = −0.06, p = 0.707, Table 2).

**Table 2:**
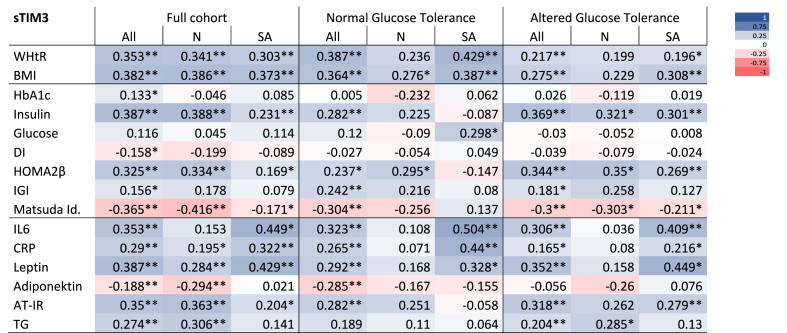
Correlation coefficients (Spearman Rho) for plasma sTIM3 and markers of metabolic health and metaflammation.

### 3.4 Soluble TIM-3 correlated with markers of metaflammation in South Asian women, and not in Nordic women

In South Asian women there was a consistent, moderate correlation in all groups between sTIM-3 and markers of metaflammation, IL-6 (r_SA_ = 0.45, p < 0.001; r_SA-NGT_ = 0.50, p < 0.001; r_SA-AGT_ = 0.41, p < 0.001), CRP (r_SA_ = 0.32, p < 0.001; r_SA-NGT_ = 0.44, p = 0.002; r_SA-AGT_ = 0.22, p = 0.023), and leptin (r_SA_ = 0.43, p < 0.001; r_SA-NGT_ = 0.33, p = 0.036; r_SA-AGT_ = 0.45, p < 0.001, Table 2). In contrast, except for a positive correlation with leptin in the Nordic women as a whole, we found no significant correlations between sTIM-3 and any of the other inflammatory markers in any of the subgroups of Nordic women (IL-6: r_N_ = 0.15, p = 0.121; r_N-NGT_ = 0.11, p = 0.428; r_N-AGT_ = 0.04, p = 0.810, CRP: r_N_ = 0.20, p = 0.047; r_N-NGT_ = 0.07, p = 0.603; r_N-AGT_ = 0.08, p = 0.587 and leptin: r_N_ = 0.28, p = 0.004; r_N-NGT_ = 0.17, p = 0.221; r_N-AGT_ = 0.16, p = 0.289).

### 3.5 Soluble TIM-3 as a possible mediator of the difference in AT-IR between ethnic groups

**Fig. 2:**
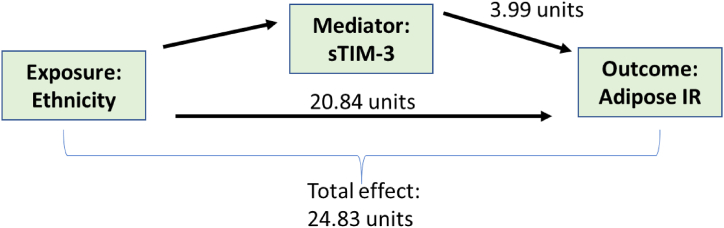
Mediation analysis in full cohort on the effect of ethnicity on AT-IR with the mediator sTIM-3. AT-IR = adipose tissue insulin resistance index, calculated as fasting insulin (pmol/L) x fasting NEFA concentrations (mmol/L).

**Fig. 3:**
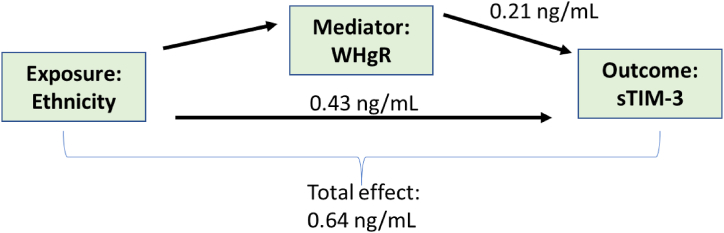
Mediation analysis in whole cohort on the effect of ethnicity on sTIM-3 concentration with WHtR as mediator. WHtR = Waist-Height Ratio.

South Asian women had a higher calculated AT-IR, as compared to the Nordic women (Table 1 [28]). To explore if T cell activation could contribute to this observed difference, we performed a mediation analysis on the total cohort (Fig. 2). The total effect of ethnicity on AT-IR was an increase in 24.83 from Nordic to South Asian ethnicity (p < 0.001). sTIM-3 mediated 16 % (p < 0.05) of this increase (Supplemental Table 1). Since the proportion of adipose tissue is thought to contribute to T cell activity, we performed a mediation analysis to explore this association (Fig. 3). The total effect of ethnicity on sTIM-3 concentration was an increase in 0.64 ng/mL (p < 0.01) from Nordic to South Asian women. A third (33 % p < 0.01) of this increase could be attributed to WHtR (Supplemental Table 2).

### 3.6 Increased plasma concentrations of T cell activation markers sCD25, sCD27 and sLAG3 in normoglycemic South Asian women after gestational diabetes

To explore if other T cell markers were regulated between the populations, we measured three other markers of T cell activation/exhaustion, i.e., sCD25, a soluble form of the α-subunit of the IL-2 receptor [29], sLAG-3, a check point inhibitor that is highly expressed by exhausted T cells [30], and sCD27, a member of the tumor necrosis receptor superfamily that is involved in persistent T cell activation [31], in the cohort. Both sCD27 and sLAG3, but not sCD25, were increased in South Asian women as compared to Nordic women (Supplemental Table 3). When dividing the cohort by glycaemic status, sCD27 showed the same patterns as sTIM-3 and was significantly increased in South Asian women as compared to Nordic women in the NGT group but not the AGT group. On the opposite, sLAG3 was significantly increased in South Asian women with AGT, compared to Nordic women; with no significant difference in the NGT group. sCD25 was, like sTIM-3 and sCD27, increased in the South Asian women in the NGT group, but reduced in South Asian women, as compared to Nordic, in the AGT group (Supplemental Table 3). As seen for sTIM-3; sCD25, sCD27 and sLAG-3 correlated positively with WHtR and BMI in South Asian women with NGT, with weaker correlation coefficients in Nordic women (Supplemental Table 4).

## 4 Discussion

South Asian women have increased risk of both GDM [32] and T2DM [33], but the mechanisms for this increased risk are not completely understood. Herein we measured plasma levels of sTIM-3 as a marker of T cell activation/exhaustion, in a cohort of women 12–36 months after GDM. We demonstrate that women from South Asia with residency in Norway have increased levels of sTIM-3 as compared to women with Nordic background, driven by the higher sTIM-3 concentrations in South Asian women with NGT when compared to Nordic women with NGT. Further, additional markers of T cell activation/exhaustion, sLAG3 and sCD27, were increased in South Asian as compared with Nordic women, and for CD27 (and similar to sTIM-3), this increase was restricted women with NGT; further supporting enhanced T cell activation/exhaustion following GDM in South Asian women.

We have previously shown that South Asian women as a group display altered glucose metabolism during OGTT while categorized as normoglycemic [3], revealing that these individuals show signs of insulin resistance not found in comparable Nordic women. Since adipose tissue function seems to be especially important for development of insulin resistance in South Asians [4,28] we suggest that markers of inflammation and immune activation, including sTIM-3, can potentially indicate the involvement of T cells in the pathogenesis of this condition in South Asian NGT women. Interestingly, we observed a different correlation pattern between sTIM-3 and markers of metabolism and metaflammation in the two ethnicities. Thus, in the South Asian women only, sTIM-3 correlated strongly with markers of metaflammation, IL-6, CRP, and leptin; correlations that were not found in the Nordic women. This may suggest an inflammatory phenotype in South Asian women following GDM that involves persistent T cell activation. However, the clinical implications of this pattern are so far elusive. In particular, although the fact that most of the differences in sTIM-3 between South Asian and Nordic women were found in those with NGT could reflect an early involvement of T cell activation in the development of T2DM in these women, the lack of such differences in those with AGT speak against such a hypothesis.

In our mediation analysis the results pointed to a small but significant contribution of sTIM-3 to the difference in AT-IR between ethnicities. Further, our analysis showed that some of this effect could be due to the differences in WHtR, suggesting that T cells may react differently to increased adipose tissue in South Asian and Nordic women. Differences in sTIM-3 between ethnicities were still significant after adjusting for WHtR or AT-IR in the population as a whole; however, only after adjusting for AT-IR in the NGT group. Thus, this supports the notion that sTIM-3 and central ethnicity/adiposity/insulin resistance is interlinked. One could hypothesize that the pathogenic effect of “sick” adipose tissue on glucose metabolism partly could be mediated by T cell activation/exhaustion. The role of T cell activation in these processes was further supported by measuring additional T cell activation markers. Thus, sLAG-3 and sCD27 were regulated in South Asian women as compared with Nordic women and for sCD25 and sCD27 increased in those with NGT. The fact that the different T cell markers, to some degree behaved differently with regards to both expression and correlation patterns is to be expected as they are all the results of different cleaving processes and different stimuli and reflect different aspects of T cell activation/exhaustion. These findings also illustrate the complexity in assessing the interaction between T cell activation/exhaustion and glucose metabolisms, and that the measurement of more than one marker is needed. Although interesting from a mechanistic point of view, the large variability in these mediators argue against utility of these markers as prediction markers in the clinical setting.

There are some other studies that points to ethnic differences in T cell activation. In one US study on breast tumours in black and white women, they found that black women had a higher CD8^+^ cytotoxic T cell density, pointing to distinct mechanisms of immune cell action between ethnicities [34]. Further, a Dutch study found ethnic differences in immune transcriptome, with increased expression of the T cell exhaustion marker lymphocyte activation gene 3 in female South Asians with T2DM, as compared to Caucasian women with T2DM [35]; further supporting a role for T cell activation in T2DM. How these ethnical differences effect disease progression is however still elusive.

This descriptive study has limitations as it cannot prove causality and in more general terms, correlations do not indicate causal relationship. Diet information was not included, neither were information on physical activity. Both have an impact on glucose metabolism and inflammation. Further, it would have been preferable to analyse expression of the actual T cell markers not only in their soluble forms in plasma but also on T cells, both on circulating and on adipose tissue resident T cells to establish the degree of correspondence between surface expression and plasma concentration in nascent and established insulin resistance. Further, although our data may support a role for T cell activation/exhaustion in the pathophysiology underlying disease; the clinical implications are still elusive.

In conclusion, we found increased levels of sTIM-3, as well as additional markers of T cell activation/exhaustion, in a population of normoglycemic South Asian women with previous gestational diabetes as compared to women of Nordic descent. Further, we found a distinct pattern of correlations between sTIM-3 *and* markers of metaflammation and insulin sensitivity, especially in women with NGT. The possible causal relationship between T cell activation and metabolic dysfunction in high-risk South Asian women is however still elusive and merits further investigation.

## CRediT authorship contribution statement

**Helene Grannes:** Writing – review & editing, Writing – original draft, Visualization, Investigation, Formal analysis. **Archana Sharma:** Writing – review & editing, Formal analysis. **Anita Suntharalingam:** Writing – review & editing, Formal analysis. **Annika E. Michelsen:** Writing – review & editing, Formal analysis, Data curation. **Pål Aukrust:** Writing – review & editing, Writing – original draft, Conceptualization. **Thor Ueland:** Writing – review & editing, Formal analysis. **Kåre I. Birkeland:** Writing – review & editing, Supervision, Investigation, Conceptualization. **Ida Gregersen:** Writing – review & editing, Writing – original draft, Investigation. **Sindre Lee-Ødegård:** Writing – review & editing, Formal analysis. **Bente Halvorsen:** Writing – review & editing, Writing – original draft, Conceptualization.

## Availability of data and material

All data generated or analyzed during this study are included in this published article and its supplementary information.

## Ethics approval and consent to participate

The study protocols were approved by the Regional Committee for Medical and Health Research Ethics, with reference number: 2018/689. The study confirms the principles outlined in the Declaration of Helsinki for use of human samples or subjects. Signed informed consent was obtained from all participants.

## Funding

This study was funded by the 10.13039/501100006095South-Eastern Norway Regional Health Authority through grants 2021071 and 2002047. The study funder was not involved in study design, collection, analysis nor interpretation of the data.

## Declaration of competing interest

The authors declare that they have no competing interests.
