# Supplementary material for: Plasma soluble TIM-3 is increased in normoglycemic South Asian women compared to Nordic women after gestational diabetes mellitus and associated with markers of metaflammation
Source: Heliyon. 2024 Nov 16;10(22):e40339. doi: 10.1016/j.heliyon.2024.e40339 (PMC11617222; doi:10.1016/j.heliyon.2024.e40339)
Supplement: Multimedia component 1 [file mmc1.docx]

**Supplemental tables**

**Supplemental Table 1**. Output from mediation analysis Ethnicity on Adipose IR via sTIM3.

|  | Estimate | Stdr Error | Z | P | Lower | Upper |
| --- | --- | --- | --- | --- | --- | --- |
| Cde | 20.8 | 4.8 | 4.4 | 0.000 | 11.5 | 30.2 |
| Pnde | 20.8 | 4.8 | 4.4 | 0.000 | 11.5 | 30.2 |
| Tnie | 4.0 | 1.54 | 2.6 | 0.009 | 1.0 | 7.0 |
| Te | 24.8 | 4.8 | 5.2 | 0.000 | 15.5 | 34.2 |
| pm | 0.16 | 0.06 | 2.5 | 0.012 | 0.04 | 0.29 |

Cde=Controlled direct effect, Pnde=Natural direct effect, Tnie=Natural Indirect effect, Te=Total effect, pm=proportion mediated.

**Supplemental Table 2.** Output from mediation analysis ethnicity on sTIM3 via WHtR.

|  | Estimate | Stdr Error | Z | P | Lower | Upper |
| --- | --- | --- | --- | --- | --- | --- |
| Cde | 0.43 | 0.17 | 2.5 | 0.012 | 0.09 | 0.76 |
| Pnde | 0.43 | 0.17 | 2.5 | 0.012 | 0.09 | 0.76 |
| Tnie | 0.21 | 0.07 | 3.0 | 0.003 | 0.07 | 0.35 |
| Te | 0.64 | 0.18 | 3.6 | 0.000 | 0.29 | 0.99 |
| pm | 0.33 | 0.12 | 2.7 | 0.007 | 0.09 | 0.57 |

|  | **All** | | | **Normal Glucose Tolerance** | | | **Altered Glucose Tolerance** | | |
| --- | --- | --- | --- | --- | --- | --- | --- | --- | --- |
|  | Nordic | South Asian | p | Nordic | South Asian | P | Nordic | South Asian | p |
| **sTIM3 (ng/ml)** | 5.00 (1.36 ) | 5.62 (1.44) | <0.001 | 4.63 (1.22) | 5.32 (1.40) | 0.009 | 5.41 (1.41) | 5.75 (1.44) | 0.166 |
| **sLAG3 (ng/ml)** | 1.79 (0.43) | 2.00 (0.48) | <0.001 | 1.77 (0.37) | 1.92 (0.49) | 0.067 | 1.82 (0.49) | 2.03 (0.48) | 0.009 |
| **sCD25^a^ (ng/ml)** | 0.42 (0.35-0.58) | 0.46 (0.35-0.57) | 0.706 | 0.38 (0.31-0.45) | 0.49 (0.35-0.61) | 0.010 | 0.50 (0.40-0.69) | 0.46 (0.35-0.56) | 0.005 |
| **sCD27 ^a^ (ng/ml)** | 4.47 (3.65-5.14) | 4.86 (4.22-5.56) | 0.003 | 4.28 (3.56-4.97) | 4.77 (4.02-5.36) | 0.024 | 4.78 (3.90-5.52) | 4.90 (4.21-5.70) | 0.244 |

Cde=Controlled direct effect, Pnde=Natural direct effect, Tnie=Natural Indirect effect, Te=Total effect, pm=proportion mediated

**Supplemental Table 3**. Plasma concentrations for sTIM3, sLAG3, sCD25 and sCD27 in Nordic and South Asian women with Normal and Altered Glucose Tolerance

Presented as ^a^mean and SD or ^b^median (q25-q75)

**Supplemental Table 4.** Correlation coefficients (Spearman Rho) for plasma sLAG-3, sCD25 and sCD27, and markers of metabolic health and metaflammation.

N= Nordic, SA= South Asian, WHtR = Waist-Height Ratio, BMI = body mass index, DI= disposition index, HOMA2-β= Homeostasis Model Assessment -β, IGI = insulinogenic index, IL-6 = interleukin 6, CRP = C reactive protein,  AT-IR = Adipose insulin resistance *p<0.05 , **p<0.01, ***p<0.001, ^a^p<0.1. Color coding is illustrating Spearman’s Rho and ranges from 1 (deep blue) to -1 (deep red).
